# Supplementary figures and images for: Multiple Hybridization Events Punctuate the Evolutionary Trajectory of Malassezia furfur
Source: mBio. 2022 Apr 11;13(2):e03853-21. doi: 10.1128/mbio.03853-21 (PMC9040865; doi:10.1128/mbio.03853-21)

**A**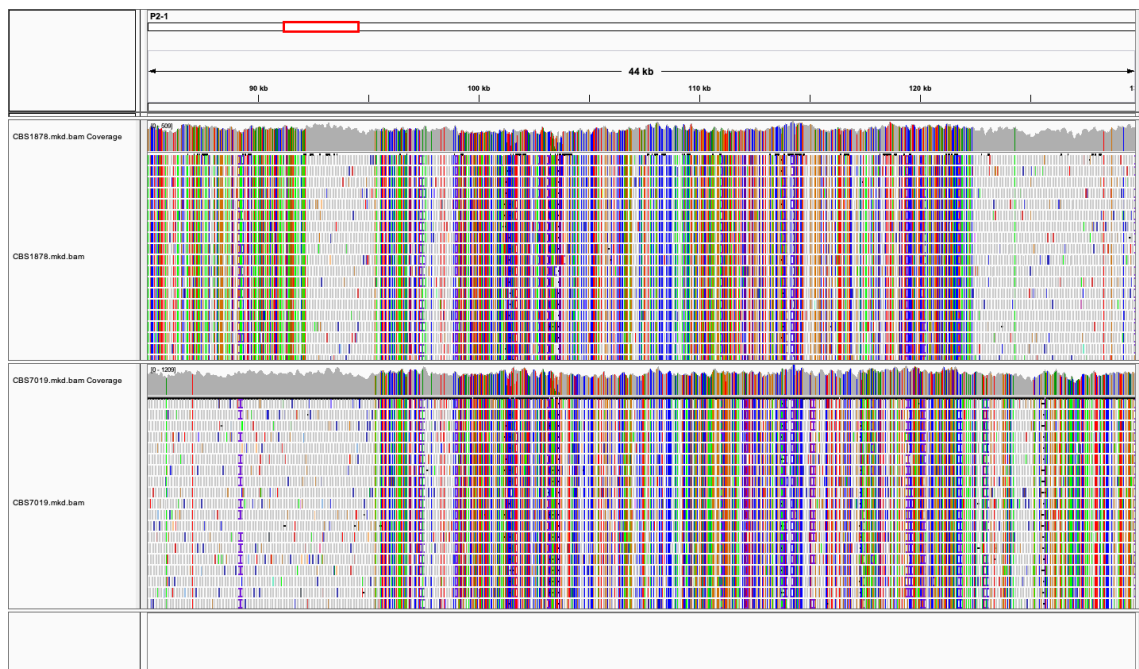**B**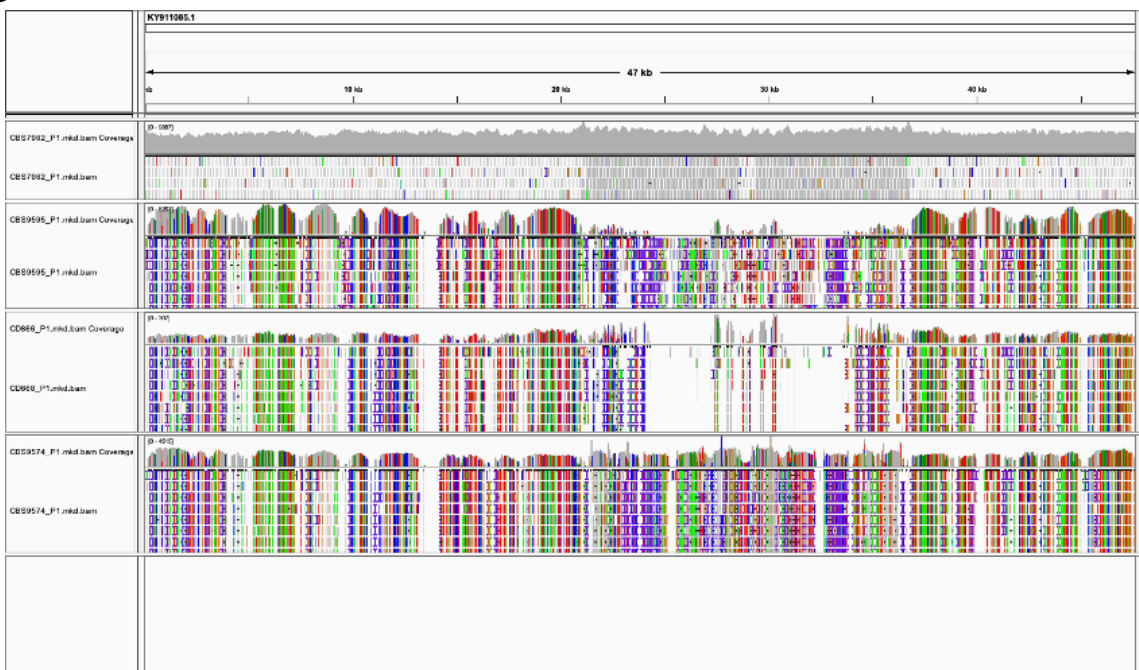**C**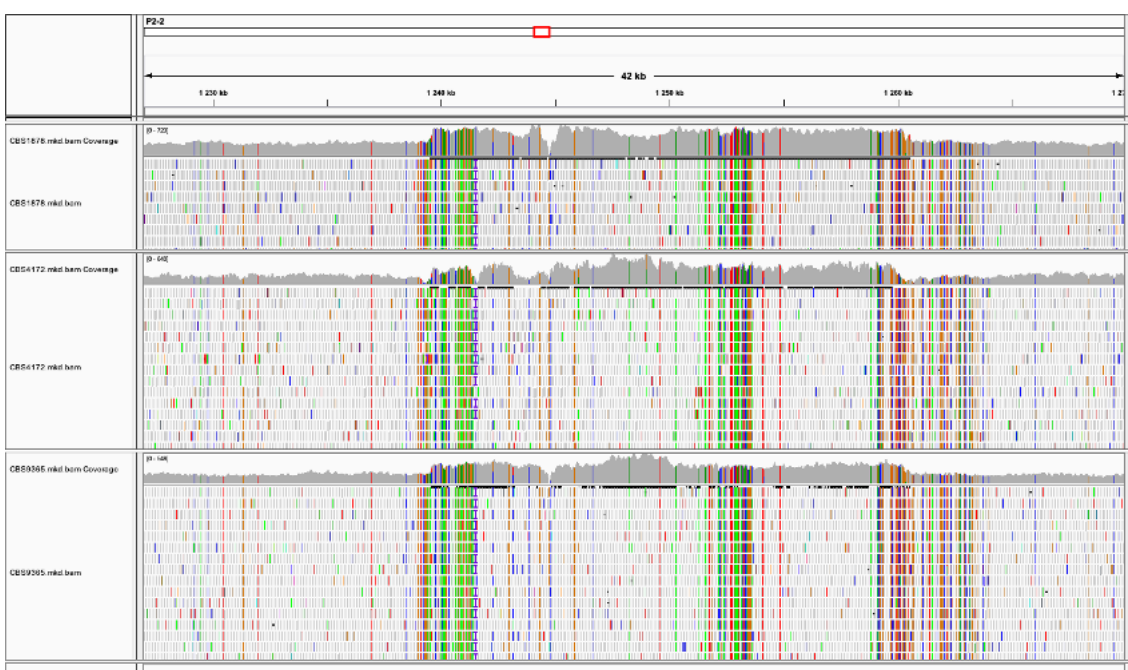

Supplement: FIG S1 [file mbio.03853-21-sf001.pdf]

A

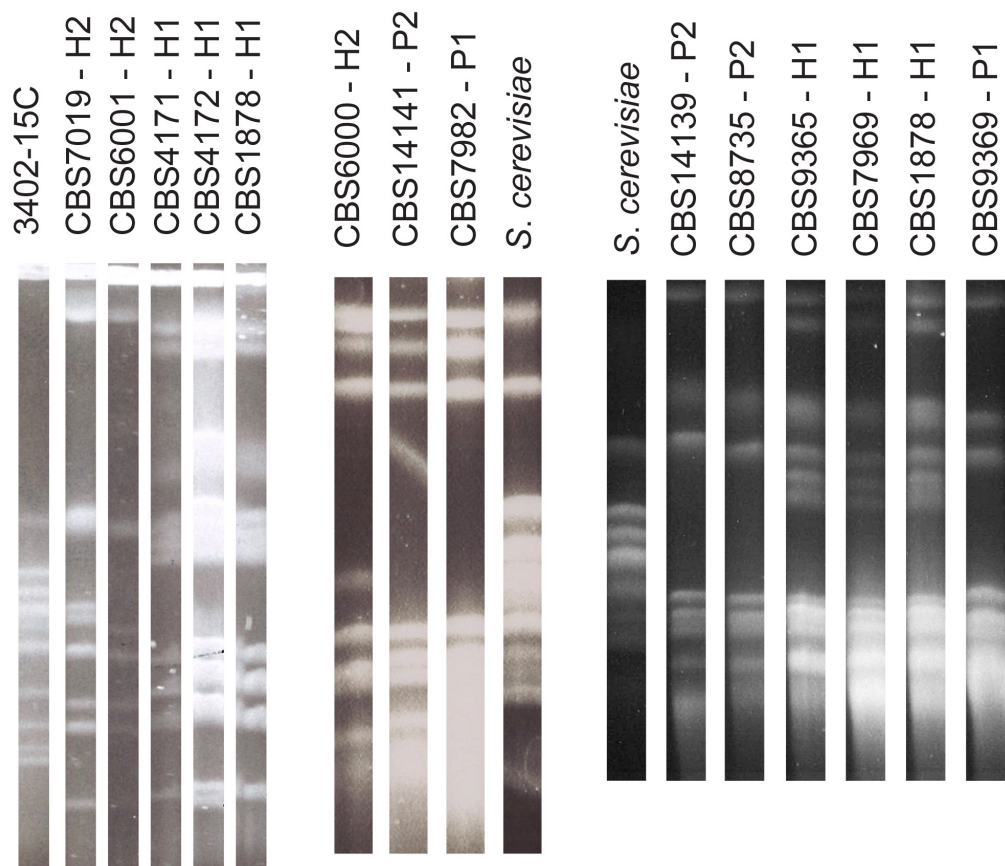

B

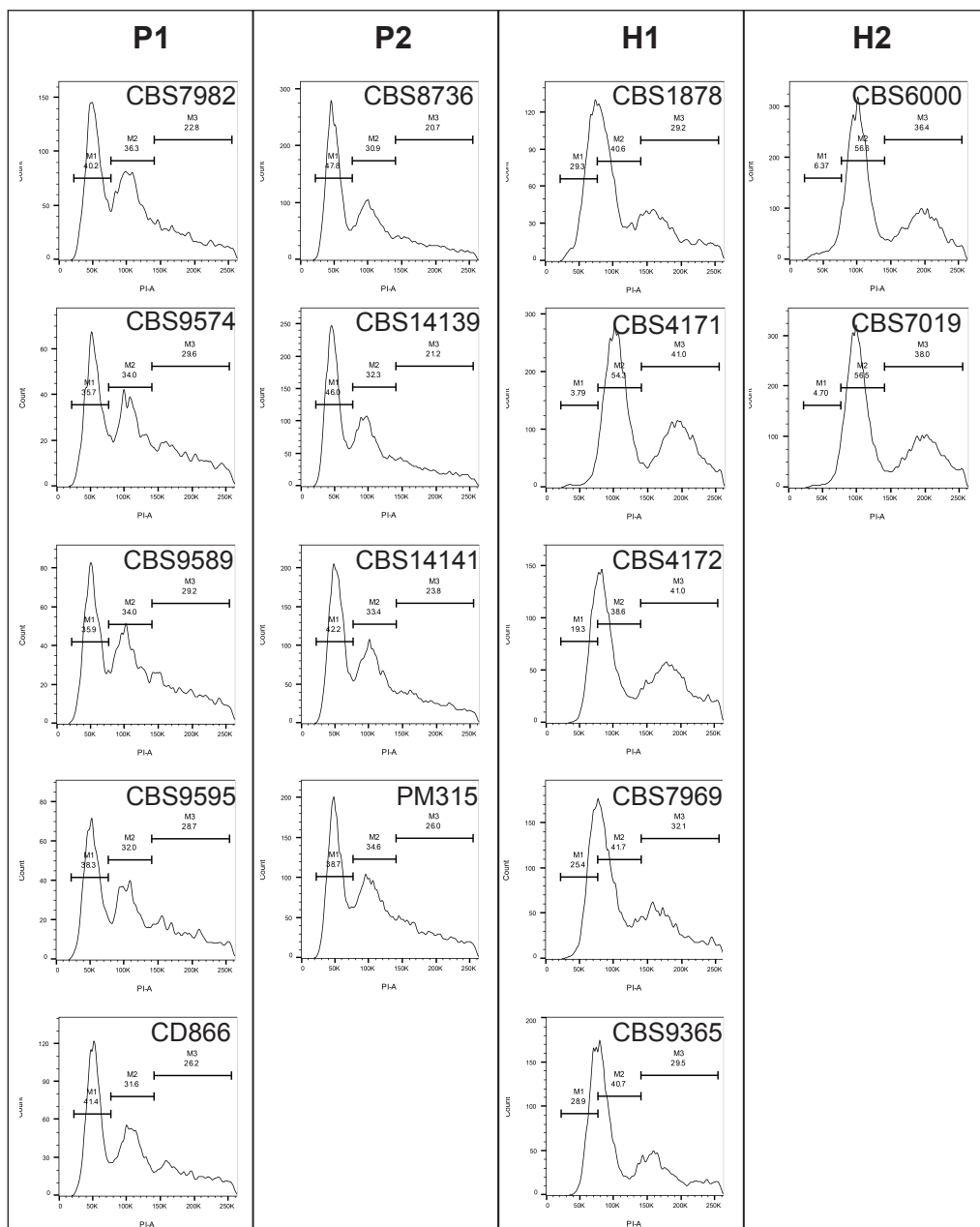

Supplement: FIG S2 [file mbio.03853-21-sf002.pdf]

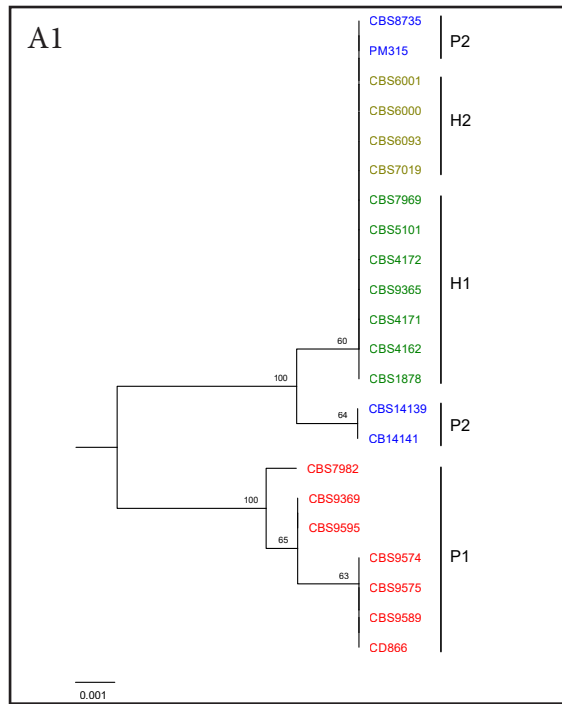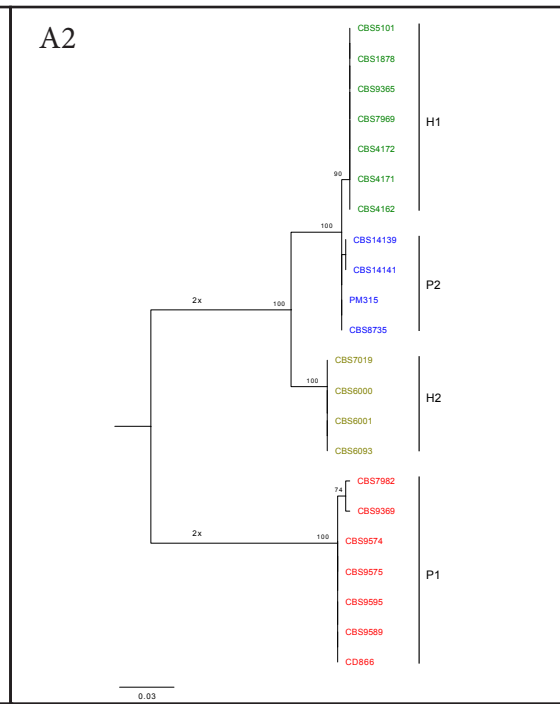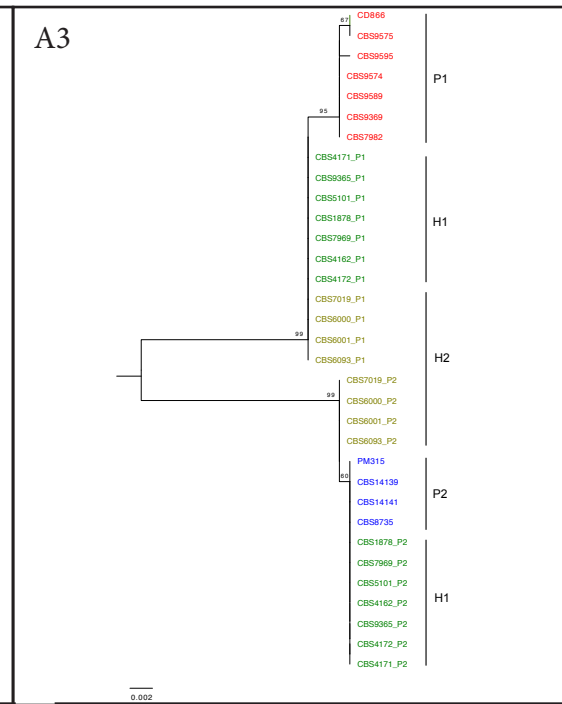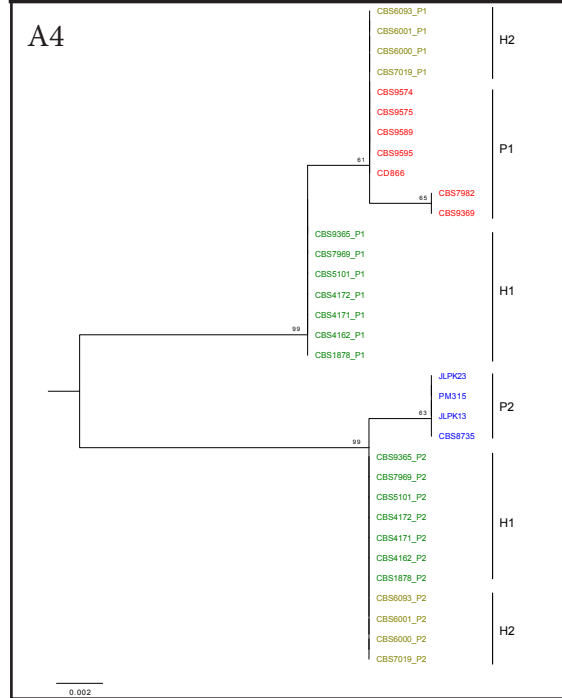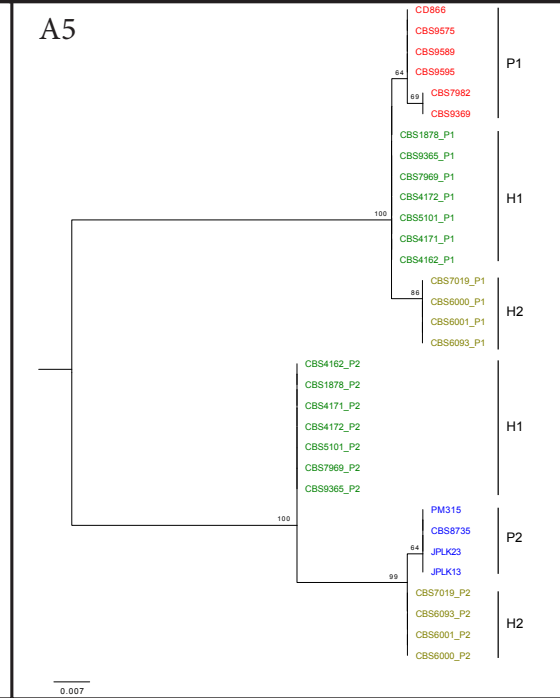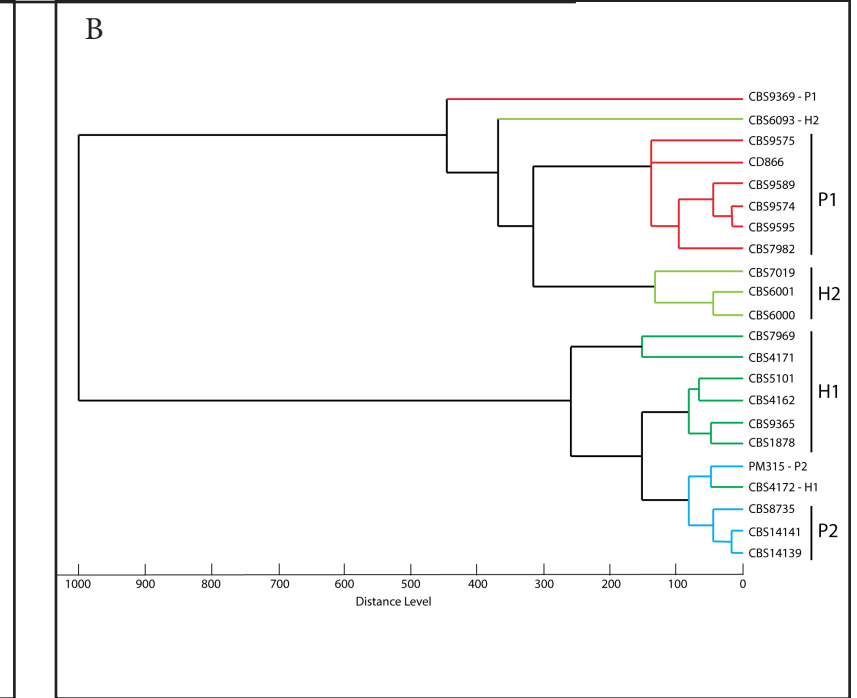

Supplement: FIG S3 [file mbio.03853-21-sf003.pdf]

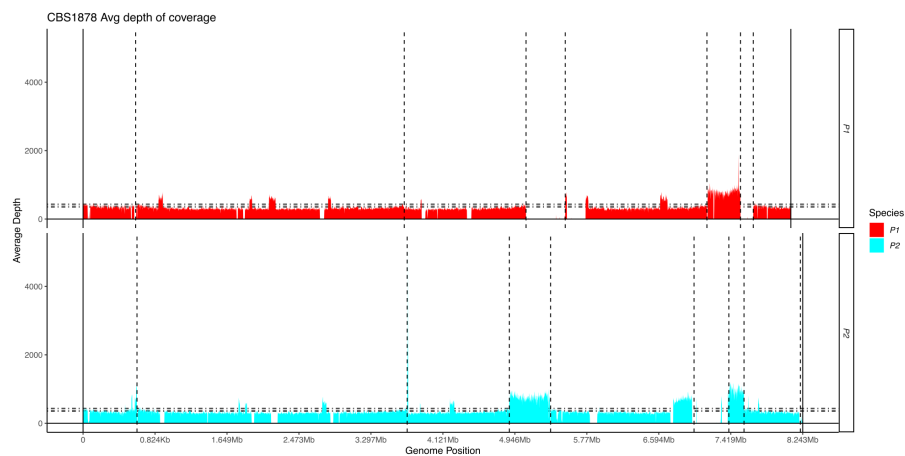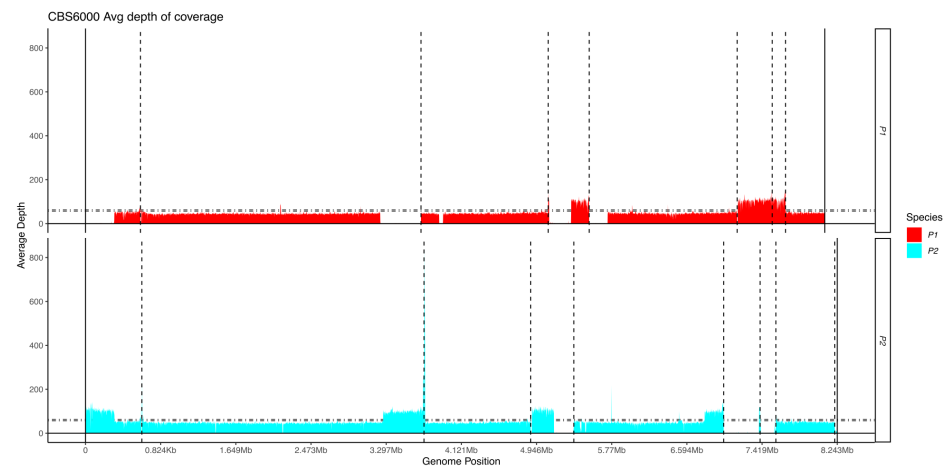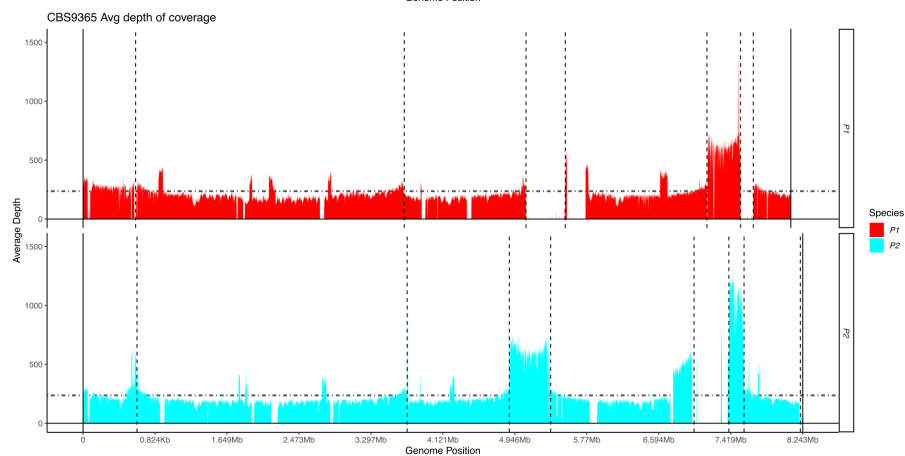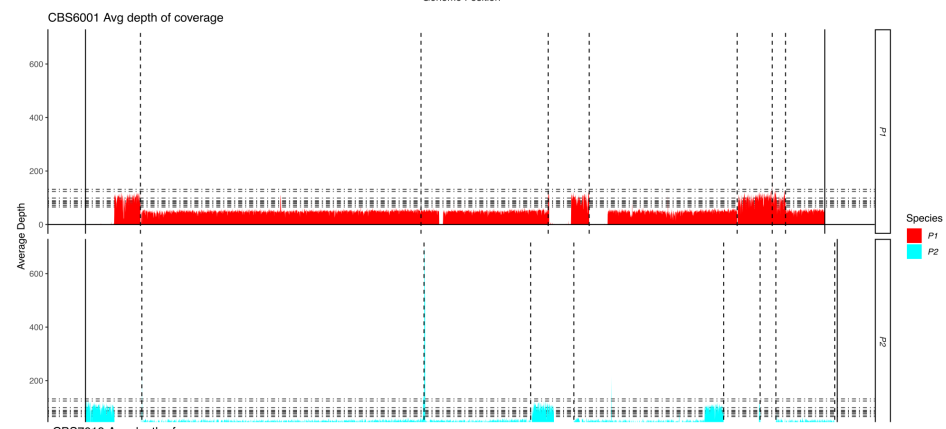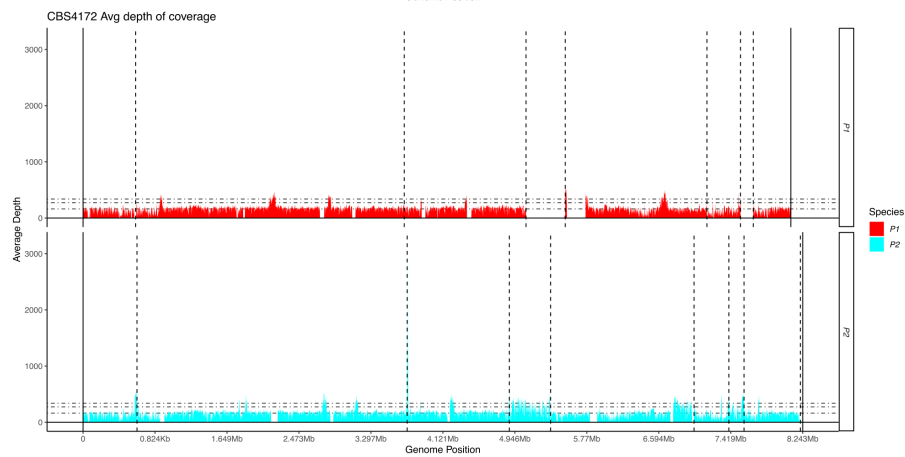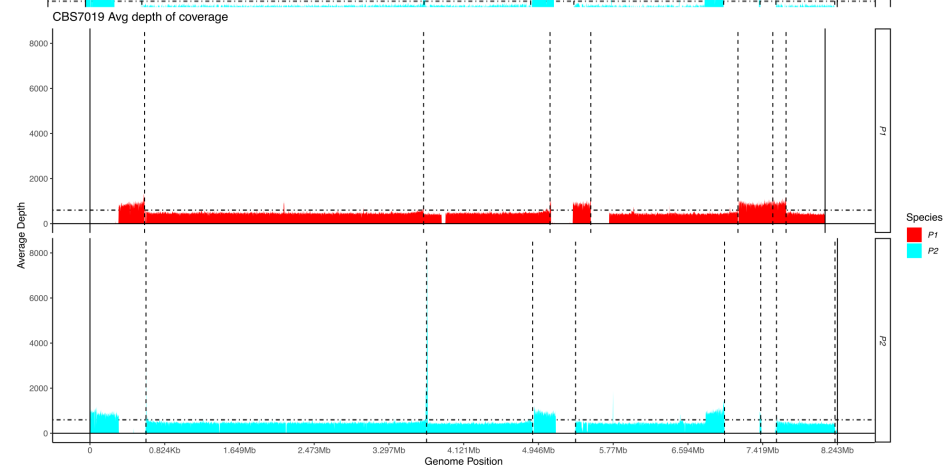

Supplement: FIG S4 [file mbio.03853-21-sf004.pdf]
